# Immunocompromise and early-onset invasive pulmonary aspergillosis in viral pneumonia: a retrospective cohort study

**DOI:** 10.3389/fpubh.2025.1723842

**Published:** 2026-01-15

**Authors:** Borui Sun, Jiechao Shen, Mengyi Dong, Yonghong Wang, Weizheng Cui, Ning Xiao, Yuehang Li

**Affiliations:** 1 Department of Pulmonary and Critical Care Medicine, Unit 1, Handan Central Hospital, Handan, China; 2 Department of Neurology, Unit 1, Handan Central Hospital, Handan, China; 3 Department of Gastroenterology, Unit 2, Handan Central Hospital, Handan, China

**Keywords:** early diagnosis, immunocompromised host, invasive pulmonary aspergillosis, outcomes, viral pneumonia

## Abstract

**Background:**

In the context of viral pneumonia, immunocompromised status represents a recognized risk factor for invasive pulmonary aspergillosis (IPA), its association with the timing of IPA diagnosis remains unclear.

**Methods:**

In the present study, 261 patients hospitalized with viral pneumonia were consecutively enrolled and categorized as immunocompromised hosts (ICHs) or non-ICHs. Baseline characteristics, outcomes, and time to IPA diagnosis were compared. Cox regression was used to evaluate the association between immunocompromised status and adverse outcomes. Patients diagnosed with IPA were further stratified into early (diagnosis within 5 days of admission) and late groups. Logistic regression was employed to evaluate the association of immunocompromised status with early-onset IPA.

**Results:**

Among the enrolled patients, 122 (46.7%) were immunocompromised. Relative to the non-ICH group, ICH patients were older, had a lower body mass index, and contained a smaller proportion of never-smokers. They also presented with higher respiratory rates, CRP, PCT, lower PaO₂/FiO₂ ratios, and greater illness severity. Significantly higher rates of invasive mechanical ventilation (20.5% vs. 2.2%), IPA incidence (22.1% vs. 9.4%), and 30-day mortality (23.8% vs. 5.0%) were observed in the ICH group compared to the non-ICH group. Multivariable Cox regression identified immunocompromised status as an independent risk factor for IPA (adjusted HR, 2.33; 95% CI, 1.07–5.06). Strikingly, immunocompromised hosts (ICHs) accounted for 80.0% of the early-onset IPA cases, compared to only 46.2% in the late-onset group. This association was confirmed in the adjusted analysis, where immunocompromised status remained a powerful independent risk factor for early diagnosis (adjusted OR 35.7, 95% CI 3.71–763.00).

**Conclusion:**

Immunocompromised status is an independent risk factor for both the development and earlier onset of IPA in patients with viral pneumonia, underscoring the need for heightened vigilance and early investigation in this high-risk population.

## Introduction

Invasive pulmonary aspergillosis (IPA) is primarily an opportunistic infection affecting immunocompromised individuals (1, 2). Established risk factors include hematopoietic stem cell transplantation (HSCT), solid organ transplantation (SOT), underlying hematologic malignancies, prolonged corticosteroid use, and neutropenia, among others (1, 3). Viral pneumonia is now well-established as a notable risk factor for IPA, with influenza and SARS-CoV-2 being the most frequently implicated pathogens (4, 5). Reflecting this understanding, the 2021 update of the EORTC/MSGERC consensus criteria incorporated severe viral pneumonia as a host factor for invasive fungal disease (6, 7).

The co-occurrence of IPA in patients with viral pneumonia is associated with worsened clinical outcomes, including higher mortality, prolonged mechanical ventilation, and extended ICU stays (8–10). Immunocompromised hosts (ICHs) with viral pneumonia are especially vulnerable, often developing severe respiratory disease with mortality rates reported between 25 and 70% (11–16). The timely diagnosis of IPA and prompt administration of antifungal agents represent a cornerstone of achieving better clinical outcomes in these patients (17, 18). Consequently, identifying high-risk individuals early and implementing intensive monitoring and aggressive management may lead to better outcomes.

While immunocompromised status is widely recognized as a risk factor for invasive pulmonary aspergillosis (IPA) in viral pneumonia (19), important questions remain regarding the nature and clinical significance of this relationship. Specifically, it is still unclear whether immunosuppression contributes to an earlier or delayed diagnosis of IPA. Defining a clear “diagnostic window” for immunocompromised patients could be crucial in guiding targeted, risk-stratified monitoring. Therefore, this study seeks to investigate whether immunocompromised status independently predicts the timing of IPA diagnosis, explore its impact on prognosis, and compare the clinical features and outcomes in patients with viral pneumonia.

## Methods

### Study population and design

This single-center, retrospective cohort study enrolled patients from a tertiary care pulmonary and critical care medicine unit. Ethical approval for this study was obtained from the Research Ethics Committee of Handan Central Hospital (Approval No.: 2025-304). A waiver of informed consent was granted in view of the retrospective design of the research.

**Figure 1:**
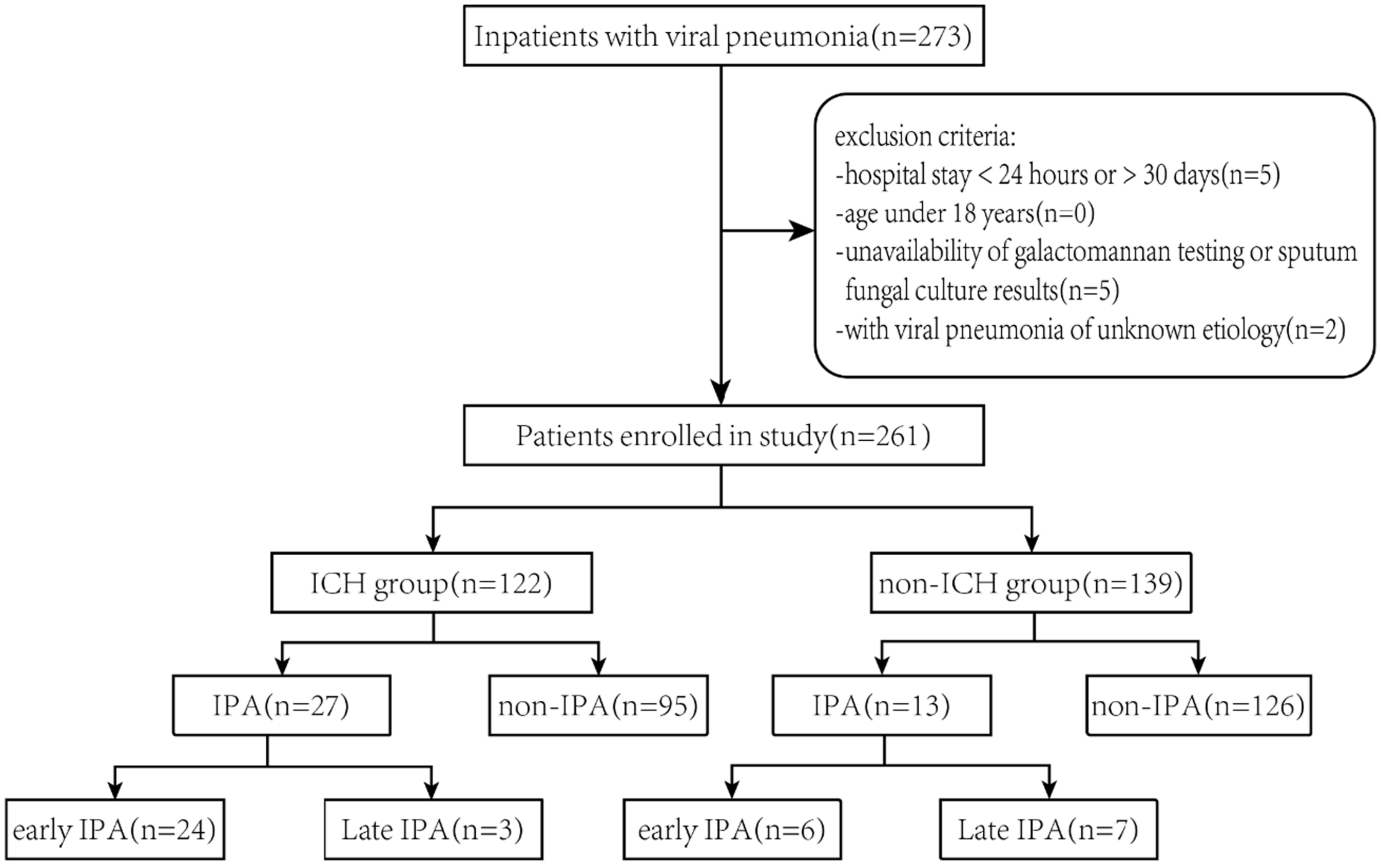
Flow diagram of eligible patients diagnosed with viral pneumonia.

The study design and patient enrollment process were illustrated in Figure 1. We consecutively enrolled hospitalized patients with viral pneumonia caused by influenza virus, SARS-CoV-2, or other respiratory viruses—specifically, cytomegalovirus (CMV), respiratory syncytial virus (RSV), or human metapneumovirus (hMPV)—between January 2020 and December 2024.

### Diagnostic criteria

The diagnosis of viral pneumonia was established based on a comprehensive assessment of clinical features, radiographic findings, and laboratory results. Each case was independently confirmed by at least three experienced physicians who were blinded to each other’s assessments.

Invasive pulmonary aspergillosis (IPA) was diagnosed according to the European Organization for Research and Treatment of Cancer and the Mycoses Study Group Education and Research Consortium (EORTC/MSGERC) criteria (6). Proven IPA was defined as: (1) histopathologic identification of hyphae morphologically compatible with Aspergillus species with evidence of associated tissue damage in a sterile specimen, necessitating confirmation by culture or PCR; or (2) culture of Aspergillus species from a sterile-site specimen (e.g., pleural effusion). Probable IPA required the concurrent presence of at least one mycological criterion, one compatible clinical/radiological abnormality, and one host factor. Mycological evidence included: a positive culture, cytology, or direct microscopy for Aspergillus from a lower respiratory tract specimen; a plasma galactomannan (GM) index >0.5; or a BALF GM index >0.8 (6).

### Exclusion criteria and definitions

Participants were excluded if they met any of the following predefined criteria: (a) hospital stay <24 h or >30 days; (b) age <18 years; (c) unavailability of galactomannan or sputum fungal culture results; or (d) viral pneumonia of unknown etiology.

Immunocompromised status was defined by meeting any of the following conditions: (a) Currently receiving high-dose (>0.5 mg/kg/day) steroids for at least 1 week or having been on long-term (>3 months) steroid therapy until disease onset; or receiving other immunosuppressant drugs. (b) History of solid organ transplantation. (c) Active solid tumor, either currently untreated or having received chemotherapy/radiotherapy within the past 5 years. (d) Diagnosis of hematologic malignancy. (e) HIV-positive status.

### Microbiological methods

Microbiological testing for all patients with viral pneumonia was performed using nasopharyngeal swabs, sputum, lower respiratory tract (LRT) specimens, or blood samples. Bronchoalveolar lavage fluid (BALF) was obtained when pathogens could not be identified by non-invasive methods.

Viral infections were identified by viral nucleic acid polymerase chain reaction (PCR), respiratory virus IgM serological testing, or an ultra-high multiplex PCR assay (Shijiazhuang Bingyuan Medical Laboratory, China). Bacterial or atypical pathogens were detected using conventional culture techniques or the aforementioned multiplex PCR assay on sputum and LRT samples.

Based on the etiological findings, study participants were categorized as follows: (a) Mono-viral pneumonia: detection of a single virus; (b) Viral co-infection pneumonia: detection of more than one virus; (c) Viral-bacterial co-infection pneumonia: concurrent detection of one or more viruses and one or more bacterial pathogens.

### Date collection and outcomes

This study utilized data derived from electronic medical records in a retrospective design. The study collected a range of variables, including demographic profiles (age, gender, BMI, smoking status, comorbidities) and initial clinical presentation, the latter comprising vital signs (body temperature, heart rate, respiratory rate, blood pressure) as well as the APACHE II score to quantify disease severity. Admission laboratory results were also analyzed, comprising complete blood count, arterial blood gas analysis, and measurements of creatinine, liver enzymes (AST, ALT), albumin, and D-dimer. Treatments and outcomes collected included: use of mechanical ventilation during hospitalization, the occurrence of invasive pulmonary aspergillosis (IPA) within 14 days of admission, and 30-day in-hospital mortality. The duration from admission until each outcome event was also recorded.

### Statistical analysis

Continuous variables were described as mean ± standard deviation or median (interquartile range, IQR), based on their distribution. Categorical variables were summarized as frequencies and percentages. The normality of continuous variables was assessed using the Kolmogorov–Smirnov test. Accordingly, group comparisons for continuous variables were performed using the Student’s *t*-test (for normally distributed data) or the Mann–Whitney U test/Kruskal-Wallis test (for non-normally distributed data). Comparisons of categorical variables were conducted using the Chi-square test or Fisher’s exact test, as appropriate. The cumulative incidence of study endpoints (e.g., invasive pulmonary aspergillosis) between immunocompromised and non-immunocompromised groups was visualized using Kaplan–Meier curves, and differences were assessed with the log-rank test. Cox proportional hazards regression models were used to analyze the time-to-event outcomes, including the onset of invasive pulmonary aspergillosis, in-hospital death, and the need for invasive mechanical ventilation. Results were expressed as hazard ratios (HRs) with 95% confidence intervals (CIs). We constructed three sequential models: Model 1 was unadjusted; Model 2 was adjusted for age, sex, and BMI; and Model 3 was further adjusted for smoking status, COPD, and APACHE II score at admission. Additionally, the association between immunocompromised status and a binary outcome of IPA diagnosis within 5 days was evaluated using logistic regression, with results presented as odds ratios (ORs) and 95% CIs. Subgroup analyses were performed to examine the consistency of the association between immunocompromised status and early-onset IPA across predefined strata: age (<75 vs. ≥75 years), sex, COPD status, smoking status, and underweight status. Interaction effects were tested using the likelihood ratio test. All statistical analyses were performed using R software (version 4.5.1; R Foundation for Statistical Computing). A two-sided *p*-value < 0.05 was considered statistically significant.

## Results

### Baseline characteristics of the cohorts

A total of 261 patients with confirmed viral pneumonia were enrolled in this study. Among them, 122 (46.7%) were classified as immunocompromised (ICH group) and 139 (53.3%) as immunocompetent (non-ICH group). The most common cause of immunocompromise was high-dose corticosteroid use (62.3%), followed by solid tumor (27.0%), hematologic malignancy (7.4%), and HIV infection (3.3%). The entire cohort had a median age of 69 years. There were 168 males (64.4%) and 93 females (35.6%). In the whole cohort, SARS-CoV-2 (73.2%) was most frequently detected, followed by influenza virus (31.8%), CMV (4.6%), RSV (2.3%), hMPV (0.8%). Based on the etiological classification, infection with a single virus accounted for the highest proportion (66.7%), followed by bacterial-viral coinfection (22.6%), viral-viral coinfection (10.7%). The proportion of patients with mixed bacterial-viral infection was higher in the ICH group than in the non-ICH group (35.2% vs. 11.5%). During the 30-day follow-up, 37 patients died, 40 were diagnosed with invasive pulmonary aspergillosis (IPA), and 28 required invasive mechanical ventilation. As shown in Table 1, the ICH group was significantly older [median (IQR): 72 (63.0–79.0) vs. 67 (55.0–74.5) years; *p* < 0.001] and had a lower BMI [18.6 (17.7–21.7) vs. 21.9 (18.6–24.0); *p* < 0.001] than the non-ICH group. Additionally, the proportion of never-smokers was higher in the non-ICH group (66.9% vs. 50.8%, *p* = 0.012). No significant gender differences or disparities in major comorbidities were found between the groups. Regarding clinical severity, the ICH group presented with significantly higher respiratory rates [median (IQR): 21 (20–24) vs. 20 (20–22) breaths/min, *p* = 0.003], CRP [median (IQR): 58.5 (21.1–115.6) vs. 37.4 (11.2–86.4) mg/L, *p* = 0.02], PCT [median (IQR): 0.12 (0.05–0.33) vs. 0.07 (0.01–0.14) ng/mL, *p* < 0.001] and APACHE II scores [median (IQR): 12 (10–15) vs. 7.5 (5–10), *p* < 0.001], along with significantly lower PaO₂/FiO₂ ratios [median (IQR): 200 (148–307) vs. 312 (270–365), *p* < 0.001] and serum albumin levels (mean ± SD: 32.9 ± 5.13 vs. 35.5 ± 5.23 g/L, *p* < 0.001). Other vital signs and laboratory parameters showed no statistically significant differences.

**Table 1:** Characteristics and outcomes of patients categorized by immunocompromised status.

Categories	Overall (*n* = 261)	ICH (*n* = 122)	Non-ICH (*n* = 139)	*p* value
Age, years	69 (58.0–77.0)	72 (63.0–79.0)	67 (55.0–74.5)	<0.001
Sex				0.70
Men	168 (64.4)	80 (65.6)	88 (63.3)	
BMI	21.1 (18.0–23.8)	18.6 (17.7–21.7)	21.9 (18.6–24.0)	<0.001
Smoking status				0.002
Never smoker	155 (59.4)	62 (50.8)	93 (66.9)	
Former smoker	67 (25.7)	44 (36.1)	23 (16.5)	
Current smoker	39 (14.9)	16 (13.1)	23 (16.5)	
Comorbidity				
Hypertension	117 (44.8)	55 (45.1)	62 (44.6)	0.94
Diabetes mellitus	74 (28.4)	39 (32.0)	35 (25.2)	0.22
COPD	29 (11.1)	18 (14.8)	11 (7.9)	0.08
Coronary heart diseases	57 (21.8)	29 (23.8)	28 (20.1)	0.48
Cerebrovascular diseases	41 (15.7)	19 (15.6)	22 (15.8)	0.96
Chronic kidney disease	26 (10.0)	13 (10.7)	13 (9.4)	0.73
Chronic liver disease	20 (7.7)	9 (7.4)	11 (7.9)	0.87
Type of immunocompromised				
Solid tumor		33 (27.0)		
Hematologic malignancy		9 (7.4)		
HIV infection		4 (3.3)		
High dose corticoid use		76 (62.3)		
Causative pathogens				<0.001
Mono-viral pneumonia	174 (66.7)	68 (55.7)	106 (76.3)	
Viral/viral co-infection	28 (10.7)	11 (9.0)	17 (12.2)	
Viral/bacterial co-infection	59 (22.6)	43 (35.2)	16 (11.5)	
Clinical characteristics at admission				
Fever (>37.3°C)	67 (25.7)	30 (24.6)	37 (26.6)	0.71
Respiratory rate, breaths per minute	20 (20–23)	21 (20–24)	20 (20–22)	0.003
Heart rate, beats per minute	85 (77–96)	86 (78–103)	84 (76–94)	0.24
Systolic blood pressure, mmHg	131 (119–143)	131 (118–143)	131 (119–143)	0.98
Diastolic blood pressure, mmHg	77 (72–86)	77 (71–84)	78 (72–88)	0.17
PaO_2_/FiO_2_	289 (200–350)	200 (148–307)	312 (270–365)	<0.001
CRP, mg/L	46.1 (18.9–102.1)	58.5 (21.1–115.6)	37.4 (11.2–86.4)	0.02
PCT, ng/mL	0.08 (0.05–0.21)	0.12 (0.05–0.33)	0.07 (0.01–0.14)	<0.001
IL-6, ng/mL	35.1 (14.2–107.5)	44.4 (18.3–147.4)	30.0 (10.8–83.4)	0.06
White blood cell count, 10^9/L	7.3 (5.4–10.0)	7.4 (5.2–10.9)	7.1 (5.6–9.7)	0.86
Albumin, g/L	34.3 ± 5.34	32.9 ± 5.13	35.5 ± 5.23	<0.001
APACHE II	10 (7–13)	12 (10–15)	7.5 (5–10)	<0.001
Ventilation				<0.001
Non-invasive ventilation	11 (4.2)	10 (8.2)	1 (0.7)	
HFNC	43 (16.5)	31 (25.4)	12 (8.6)	
Invasive ventilation	28 (10.7)	25 (20.5)	3 (2.2)	
Outcome				
30-day in-hospital death	36 (13.8)	29 (23.8)	7 (5.0)	<0.001
IPA diagnosed in 30 days	40 (15.3)	27 (22.1)	13 (9.4)	0.004
Interval to IPA diagnosis, days	4 (3–7)	4 (2–5)	7 (4–8)	0.032
Length of stay, days	9 (7–13)	11.5 (8–16)	9 (7–12)	<0.001

Data are presented as n (n%), median(IQR) and mean ± sd. ICH, immunocompromised host; BMI, Body Mass Index; COPD, chronic obstructive pulmonary disease; HIV, human immunodeficiency virus; APACHE II, acute physiology and chronic health evaluation II; HFNC, high flow nasal cannula; IPA, invasive pulmonary aspergillosis.

### Ventilatory support and clinical outcomes

The immunocompromised (ICH) group required higher levels of respiratory support compared to the immunocompetent (non-ICH) group. The utilization of all forms of respiratory support was significantly greater in the ICH group (all *p* < 0.05), including invasive ventilation (20.5% vs. 2.2%), HFNC (25.4% vs. 8.6%), and NIPPV (8.2% vs. 0.7%). Within the ICH group, 45.9% of patients were managed with low-flow nasal cannula or room air alone, without advanced ventilatory support. Among those who required support, HFNC was the most common modality (25.4%), followed by invasive ventilation (20.5%) and NIPPV (8.2%). Clinical outcomes were notably worse in the ICH group. Immunocompromised patients experienced significantly higher 30-day in-hospital mortality (23.8% vs. 5.0%, *p* < 0.001). The incidence of invasive pulmonary aspergillosis (IPA) was also notably higher in this group (22.1% vs. 9.4%, *p* = 0.004). Moreover, among patients diagnosed with IPA, the median time from admission to diagnosis was shorter in the ICH group (4 days, IQR 2–5) than in the non-ICH group (7 days, IQR 4–8; *p* = 0.032). The median hospital length of stay was also longer in the ICH group (11.5 days, IQR 8–16) compared to the non-ICH group (9 days, IQR 7–12, *p* < 0.001).

### Immunocompromised status and adverse outcomes

**Figure 2:**
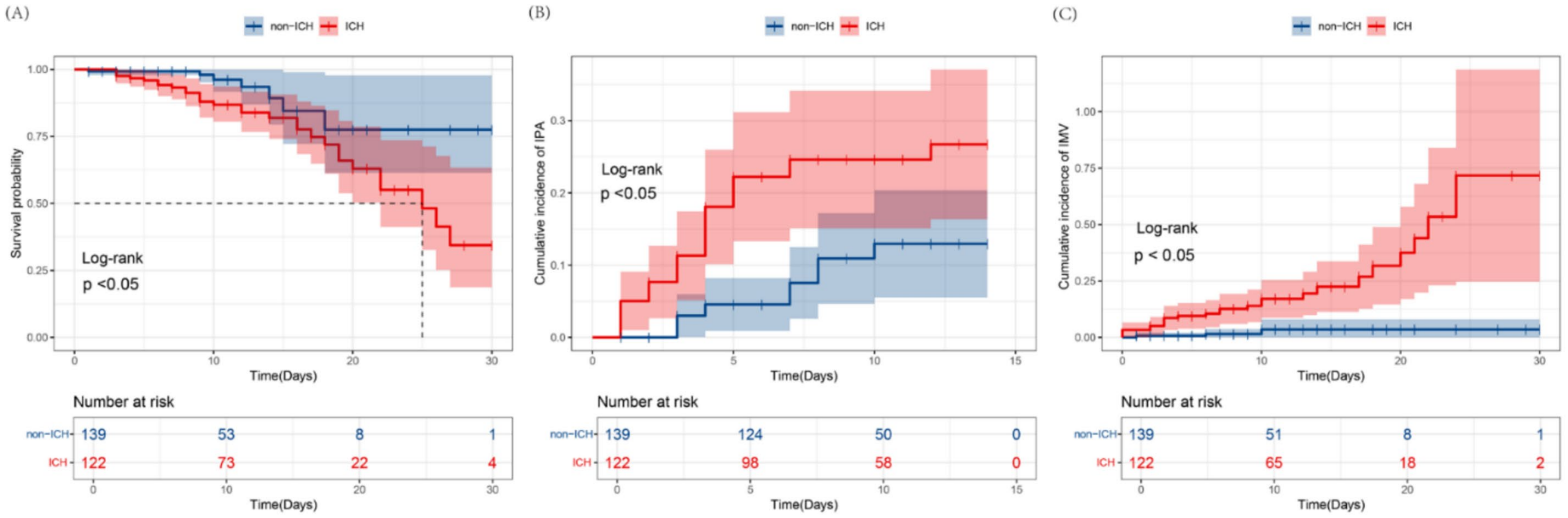
Association between immunocompromised status and clinical outcomes. Kaplan–Meier curves for 30-day mortality **(A)** and cumulative incidence curves for invasive pulmonary aspergillosis (IPA) within 14 days **(B)** and invasive mechanical ventilation (IMV) within 30 days **(C)** are compared between immunocompromised (ICH) and immunocompetent (non-ICH) patients using the log-rank test. ICH, Immunocompromised host; IMV, invasive mechanical ventilation; IPA, invasive pulmonary aspergillosis.

Significant differences in clinical outcomes were observed between immunocompromised (ICH) and immunocompetent patients via Kaplan–Meier analysis. The ICH group showed a higher cumulative incidence of 30-day mortality, IPA diagnosis within 14 days, and IMV requirement within 14 days (log-rank test, all *p* < 0.05; Figures 2A–C). In the univariable analysis (Model 1), immunocompromised status significantly increased the risk of 30-day mortality (HR 2.83, 95% CI 1.22–6.55), IPA within 14 days (HR 2.47, 95% CI 1.27–4.79), and IMV requirement (HR 7.73, 95% CI 2.32–25.77). These associations persisted after adjustment for age, sex, and BMI in Model 2. However, in the fully adjusted model (Model 3), which further included smoking status, COPD, and APACHE II score, immunocompromised status remained independently associated only with IPA diagnosis (HR 2.33, 95% CI 1.07–5.06). The associations with 30-day mortality and IMV requirement, however, were attenuated and lost statistical significance after these additional adjustments (Table 2).

**Table 2:** COX hazard regression analysis for association between immunocompromised status and outcomes.

Outcomes	Model 1		Model 2		Model 3	
	HR (95%CI)	*p* value	HR (95%CI)	*p* value	HR (95%CI)	*p* value
In-hospital death	2.83 (1.22–6.55)	0.015	2.46 (1.07–5.67)	0.035	1.62 (0.63–4.14)	0.316
IPA within 14 days	2.47 (1.27–4.79)	0.007	2.56 (1.28–5.12)	0.008	2.33 (1.07–5.06)	0.032
IMV	7.73 (2.32–25.77)	<0.001	6.09 (1.82–20.41)	0.003	3.45 (0.94–12.7)	0.062

IPA, invasive pulmonary aspergillosis; IMV, invasive mechanical ventilation; HR, hazard ratio; CI, confidence interval; Model 1, unadjusted; Model 2, adjusted for age, gender, BMI; Model 3, adjusted for age, gender, BMI, Smoking status, COPD and APACHE II.

### Immunocompromise status was associated with early diagnosis of IPA

The 40 patients diagnosed with invasive pulmonary aspergillosis (IPA) were divided into groups according to the time from admission to diagnosis, to investigate whether immunocompromised status (ICH) was linked to earlier presentation. The classification of IPA into early- and late-onset was based on a 5-day threshold from hospital admission. Immunocompromised hosts constituted a markedly larger share of the early-onset IPA group relative to the late-onset group (80.0% vs. 46.2%, respectively; *p* < 0.05). Other measured parameters, including the remaining baseline characteristics, ventilation status, and 30-day survival, did not differ significantly between the early and late IPA groups. Immunocompromised status was a strong and independent predictor of early-onset IPA. The magnitude of this effect was substantial in both unadjusted (OR 9.33, 95% CI 1.99–54.90) and adjusted analyses (aOR 14.8, 95% CI 2.52–133.00), confirming its importance. Further adjustment for smoking status, COPD, and APACHE II score, this association maintained (fully adjusted OR, 35.7; 95% CI, 3.71–763.00; *p* < 0.05) (see Table 3).

**Table 3:** Characteristics and outcomes of patients diagnosed of IPA categorized by interval to diagnosis.

Characteristics	Overall (*n* = 40)	Early IPA (*n* = 30)	Late IPA (*n* = 10)	*p* value
Age, years	69 (57.8–80.5)	68.5 (58.3–79.8)	71 (57.5–79.8)	0.94
Sex				0.43
Men	28 (70.0)	22 (73.3)	6(60.0)	
BMI	20.7 (17.3–23.7)	20.6 (17.4–23.8)	20.9 (16.5–23.2)	0.98
Smoking status				0.67
Never smoker	20 (50.0)	15 (50.0)	5 (50.0)	
Former smoker	15 (37.5)	12 (40.0)	3 (30.0)	
Current smoker	5 (12.5)	3 (10.0)	2 (20.0)	
Comorbidity				
Hypertension	20 (50.0)	13 (43.3)	7 (70.0)	0.27
Diabetes mellitus	14 (35.0)	13 (43.3)	1 (10.0)	0.13
COPD	8 (20.0)	4 (13.3)	4 (40.0)	0.17
Coronary heart diseases	5 (12.5)	3 (10.0)	2 (20.0)	0.78
Cerebrovascular diseases	6 (15.0)	4 (13.3)	2 (20.0)	0.61
Chronic kidney disease	7 (17.5)	5 (16.7)	2 (20.0)	0.73
Clinical characteristics at admission				
Fever (>37.3°C)	7 (17.5)	6 (20.0)	1 (10.0)	0.81
Respiratory rate, breaths per minute	22.3 ± 4.1	22.5 ± 4.1	21.6 ± 4.3	0.56
Heart rate, beats per minute	89 ± 17.1	90 ± 14.7	86 ± 23.6	0.63
Systolic blood pressure, mmHg	130 ± 17.5	129 ± 14.9	132 ± 24.6	0.73
Diastolic blood pressure, mmHg	77 ± 9.9	77 ± 9.3	76 ± 12.3	0.84
PaO_2_/FiO_2_	241 (198–302)	233 (172–298)	278 (236–312)	0.13
CRP	65.9 (20.7–147.2)	63.1 (20.5–140.4)	99.2 (35.5–152.9)	0.53
PCT	0.16 (0.06–0.34)	0.16 (0.06–0.29)	0.13 (0.05–0.46)	0.96
IL-6	66.9 (26.2–98.7)	81.6 (30.0–126.5)	45.7 (23.9–64.6)	0.53
White blood cell count, 10^9/L	8.7 (5.9–12.5)	7.1 (5.8–12.6)	9.2 (7.7–12.1)	0.47
Albumin, g/L	33.0 ± 5.78	33.1 ± 5.91	32.8 ± 5.68	0.88
APACHE II	11 (8–14)	11 (8–14)	10 (8–13)	0.52
ICH	27 (67.5)	24 (80.0)	6 (46.2)	0.01
Ventilation				0.62
Non-invasive ventilation	3 (7.5)	2 (6.7)	1 (10.0)	
HFNC	11 (27.5)	10 (33.3)	1 (10.0)	
Invasive ventilation	6 (15.0)	4 (13.3)	2 (20.0)	
Outcome				
30-days in hospital death	12 (30.0)	8 (26.7)	4 (40.0)	0.69
Length of stay	9 (8–14)	9 (8–13)	9 (9–14)	0.35
Interval to IPA diagnosis, days	4 (3–6)	3 (2–4)	8 (7–8)	<0.001

Early IPA, IPA diagnosed within 5 days; Late IPA, IPA diagnosed more than 5 days; ICH, immunocompromised host; BMI, Body Mass Index; COPD, chronic obstructive pulmonary disease; HIV, human immunodeficiency virus; APACHE II, acute physiology and chronic health evaluation II; HFNC, high flow nasal cannula; IPA, invasive pulmonary aspergillosis.

### Subgroup analyses

**Figure 3:**
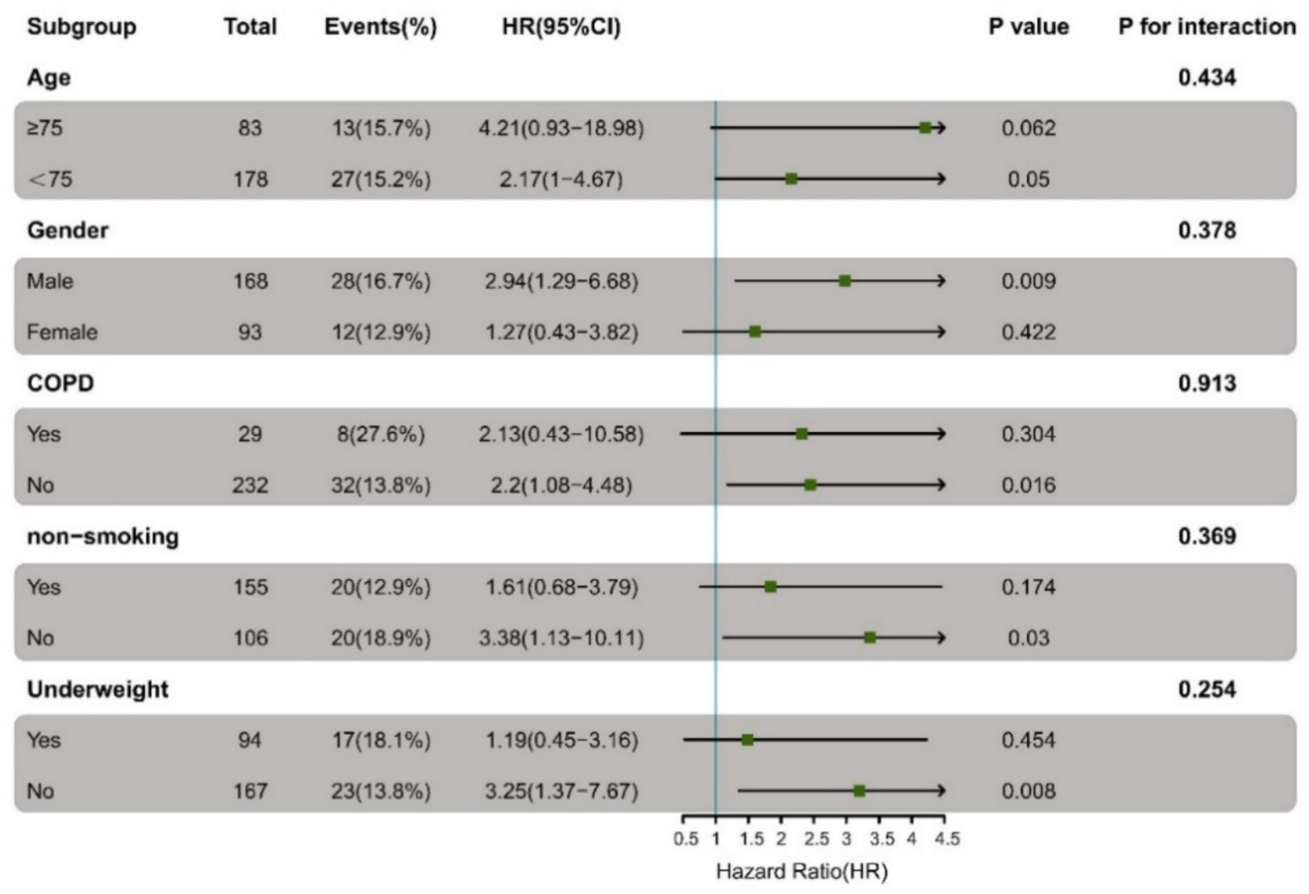
Subgroup analysis of the association between immunocompromised status and invasive pulmonary aspergillosis (IPA) onset. Associations between immunocompromised status (ICH) and incident IPA are presented as hazard ratios (HRs) with 95% CIs across predefined patient subgroups. *p* values for interaction assess the effect modification by each subgroup variable.

To assess the consistency of the immunocompromised status-IPA association, we performed subgroup analyses based on age, gender, COPD status, smoking history, and body weight. As illustrated in Figure 3, Immunocompromised patients demonstrated a consistently elevated risk of IPA across most predefined subgroups. The increased risk of IPA associated with immunocompromised status was consistent in several key subgroups, including those aged <75 years (HR 2.17), males (HR 2.94), non-COPD patients (HR 2.20), never-smokers (HR 3.38), and non-underweight individuals (HR 3.25). None of the subgrouping variables showed a significant effect modification on the ICH-IPA association (all interaction *p* > 0.05), supporting the consistency of the relationship across populations.

## Discussion

This retrospective cohort study found that immunocompromised status conferred a significantly higher risk of developing invasive pulmonary aspergillosis (IPA) in patients with viral pneumonia. Consistent with this finding, the clinical course of immunocompromised (ICH) patients was burdened by significantly higher requirements for invasive mechanical ventilation, increased occurrence of IPA, elevated 30-day mortality, and prolonged hospitalizations compared to immunocompetent patients. Importantly, after controlling for a set of predefined covariates (age, gender, BMI, smoking status, COPD, and APACHE II score), ICH remained an independent risk factor for IPA. Furthermore, our findings demonstrate that immunocompromised patients had a propensity for earlier presentation of IPA. The ICH group showed a higher rate of early IPA diagnosis, with a significantly greater proportion of cases identified within the first 5 days of admission. Even after accounting for potential confounding variables, immunocompromised status was confirmed to be an independent risk factor for early-onset IPA.

Previous studies have demonstrated that influenza virus and SARS-CoV-2 have been identified as key viral pathogens predisposing patients to invasive pulmonary aspergillosis (IPA) in the setting of viral pneumonia (4, 19, 20). Reported incidence rates of influenza-associated pulmonary aspergillosis (IAPA) range from 1.21 to 31.1%, and reported mortality between 22.2 and 100% (19, 21–25). Similarly, COVID-19-associated pulmonary aspergillosis (CAPA) has an estimated incidence of 3.3–33%. Among affected patients, mortality rates of 44–71% have been reported (4, 26–31). We observed a lower mortality from IPA in our cohort relative to that described in previous studies. A potential explanation for this discrepancy may be the generally lower severity of illness in our cohort, as reflected by lower rates of invasive mechanical ventilation and lower APACHE II scores compared to those in previous reports. Additionally, differences in outcome definitions should be considered. While prior studies often used all-cause in-hospital or ICU mortality as endpoints, our study specifically assessed 30-day in-hospital mortality. Since the median time from admission to death in some studies exceeds 30 days, our outcome definition, focusing on 30-day in-hospital mortality, could have consequently underestimated the complete mortality burden attributable to IPA.

It is well-recognized that immunocompromised status constitutes a major risk factor for invasive pulmonary aspergillosis (IPA), especially among individuals with hematologic malignancies or chemotherapy-induced neutropenia, those receiving immunosuppressive therapy for autoimmune diseases, or recipients of bone marrow or solid organ transplantation (32, 33). Among these conditions, hematological malignancies and stem cell transplantation have been identified as carrying the highest risk (34, 35). Previous studies have reported IPA incidence rates ranging from 2 to 8% in immunocompromised populations, with a trend of increase over time, potentially attributable to improved diagnostic techniques, wider use of immunosuppressive agents, and rising numbers of transplant recipients (36–40). However, an incidence of IPA as high as 51.9% was reported in a previous observational cohort of immunocompromised patients (41). In the present study, the observed incidence of IPA among immunocompromised patients was comparatively lower. This discrepancy may be explained by the distinct composition of our immunocompromised cohort, which consisted predominantly of patients on high-dose corticosteroids, followed by those with solid tumors, hematologic malignancies, and HIV infection. The relatively low proportion of individuals with hematologic malignancies or organ transplantation—groups known to be at the highest risk—may account for the lower incidence observed in our study. Further large-scale studies are warranted to more accurately delineate the epidemiology of IPA in heterogeneous immunocompromised populations.

It is widely recognized that immunocompromised hosts with viral pneumonia face substantially elevated risks of developing severe complications, with reported mortality rates as high as 25–70% (12–16, 42). Previous studies have yielded varying findings regarding the impact of immunocompromised status on outcomes. For instance, one study reported that influenza patients with an EORTC/MSG host factor had an IPA incidence of 32%, of whom 71% died—a rate substantially higher than that in immunocompetent patients (7, 19). In contrast, another study found no significant difference in disease severity or prognosis between immunocompromised and immunocompetent viral pneumonia patients (42). In the present study, immunocompromised viral pneumonia patients exhibited a significantly higher incidence of IPA (22.1% vs. 9.4%) and higher mortality (23.8% vs. 5.0%) compared to non-immunocompromised patients. Furthermore, the association between immunocompromised status and IPA remained robust across all predefined subgroups, confirming its role as an independent risk factor unaffected by variations in age, gender, smoking history, body weight, or COPD status. Larger prospective studies are warranted to further elucidate the relationship between immunocompromised status and the incidence and mortality of IPA in this patient population.

Invasive pulmonary aspergillosis (IPA) complicating viral pneumonia is associated with high mortality, underscoring the critical importance of early antifungal intervention, as highlighted in previous studies (17, 18). Bronchoscopy with bronchoalveolar lavage for mycological analysis remains a cornerstone of the diagnostic approach and should be considered in any patient with suspected IPA (3, 18). To the best of our knowledge, this study is one of the first to specifically identify an association between immunocompromised status and early-onset IPA in viral pneumonia population. However, when interpreting this finding, it is important to note that the number of early-onset IPA events was limited. Consequently, the logistic regression model, while indicating a significant association, yielded odds ratios with wide confidence intervals. This suggests that the point estimate of the effect size should be interpreted with caution regarding its precision, rather than as a stable quantitative measure. These findings raise an important clinical question: could early empirical antifungal therapy or antifungal prophylaxis be beneficial for immunocompromised patients with viral pneumonia? Additional research is warranted to establish evidence-based treatment protocols for IPA in immunocompromised hosts. It is also important to consider that the observed earlier diagnosis in the ICH group could be influenced, at least in part, by a lower clinical threshold for initiating diagnostic workups (e.g., bronchoscopy and GM testing) in these high-risk patients, in addition to any potential biological predisposition to earlier onset.

Our study has several limitations. First, its retrospective design inherently limited the sample size, and our findings require validation in larger, prospective cohorts. Second, the majority of IPA cases were clinically diagnosed due to the general unavailability of histopathological confirmation, which may have led to an overestimation. Third, due to the retrospective design, detailed immune parameters such as comprehensive cytokine profiles or lymphocyte subset counts were not routinely available, limiting the exploration of underlying immunological mechanisms. Fourth, the requirement for mycological evidence (GM/culture) and the restriction of the analysis to IPA diagnosed within 14 days of admission (while excluding hospital stays >30 days) may have introduced a selection bias by preferentially including early, suspected cases and potentially missing late-onset IPA. This was a necessary compromise to ensure diagnostic certainty and a homogeneous cohort for analyzing early diagnostic timing. Finally, the limited number of immunocompromised patients precluded a meaningful comparison across different underlying immunosuppressive conditions.

## Conclusion

This study demonstrates that immunocompromised patients with viral pneumonia are at an increased risk of invasive pulmonary aspergillosis (IPA) and are diagnosed earlier than immunocompetent patients. These results call for increased clinical awareness and prompt screening for IPA in this high-risk group.

## Data Availability

The original contributions presented in the study are included in the article/supplementary material, further inquiries can be directed to the corresponding authors.
